# CSF TNF α levels were associated with conversion from mild cognitive impairment to dementia

**DOI:** 10.1371/journal.pone.0274503

**Published:** 2022-10-26

**Authors:** Pan Fu, Feifei Peng, for the Alzheimer’s Disease Neuroimaging Initiative

**Affiliations:** Department of Neurology, Taizhou First People’s Hospital, Zhejiang, China; Nathan S Kline Institute, UNITED STATES

## Abstract

We aimed to examine the association of CSF tumor necrosis factor-alpha (TNFα) with conversion from mild cognitive impairment (MCI) to dementia. At baseline, there were a total of 129 participants with MCI in this study. The association of CSF TNFα levels with the incidence of dementia were evaluated using Cox proportional hazards regression analysis adjusted for potential confounders. Individuals were categorized into groups based on the CSF TNFα tertiles. Compared to the low group (the reference group), the intermediate group progressed more rapidly to dementia [HR (95% CI) = 2.2 (1.15–4.1); p = 0.016] after adjusting for other covariates. However, the high group did not progress faster than the low group [HR (95% CI) = 1.5 (0.79–2.8); p = 0.214]. Our study suggested a potential non-relationship between CSF TNFα levels and the risk of development of dementia among MCI older people.

## Introduction

Mild cognitive impairment (MCI) was conceptualized as a transitional disease stage between normal cognition and Alzheimer’s disease (AD) dementia more than two decades ago [1]. However, many MCI participants are either cognitively stable or convert to AD dementia or other types of dementia during several years because the MCI syndrome is heterogeneous [2]. Therefore, identifying those with MCI who will progress to AD dementia in the future is clinically important so that potential therapies can be targeted toward those who may be likely to benefit.

Emerging data indicates that inflammation plays a critical role in the pathogenesis of AD [3]. Tumor necrosis factor-alpha (TNFα) is one of the key pro-inflammatory cytokines expressed by activated microglia and astrocytes, and has been reported to be increased in the CSF of subjects with MCI and AD [4]. In a cross-sectional study, Culjak and colleagues found that serum TNFα levels were significantly higher in AD patients than in MCI subjects [5]. A variant of TNFα gene (-308A/G genotype) has been reported to be more susceptible to development of neuroinflammation, and subsequently of AD [6]. In addition, higher levels of TNFα in blood were associated with the risk of incident AD among cognitively normal community-dwelling older adults [7]. However, Diniz and colleagues did not find a link between serum TNFα levels and progression from MCI to AD during approximately 1.5 years of follow-up [8]. Similarly, Taipa and colleagues did not observe a relationship between CSF TNFα and cognitive status at baseline and follow-up in patients with AD [9]. To the best of our knowledge, among MCI participants, the association of CSF TNFα levels with the risk of conversion to dementia remains unclear.

At the present study, we aimed to investigate the association of CSF TNFα and its related receptors with the risk of developing dementia among MCI subjects.

## Materials and methods

### Alzheimer’s Disease Neuroimaging Initiative

Data used in the preparation of this work were extracted from the Alzheimer’s Disease Neuroimaging Initiative (ADNI) database. The ADNI study was initiated in 2003 with the primary goal of investigating whether neurocognitive assessments, neuroimaging markers, and other biological markers can be integrated to predict cognitive decline and clinical progression. Detailed information can be found at the website (www.adni-info.org).

### Participants

Our study focuses on the 129 participants who were diagnosed with amnestic MCI at baseline and had an initial analysis of CSF TNFα, TNFR1 and TNFR2. The criteria for MCI included the presence of a memory complaint verified by study partner, a Mini-mental state examination (MMSE) [10] score ranging from 24 to 30, a Clinical dementia rating (CDR) [11] score of 0.5, an objective memory impairment evidenced by the Logical Memory II subscale from the Wechsler Memory Scale-revised, and the presence of functional deficit not severe enough to meet the criteria for dementia. Our MCI participants were further classified into two groups (non-converters and converters) based on whether they converted to dementia during follow-up. At each ADNI site, participants provided written informed consents, and local institutional review board approved the ADNI study. For the names of all ADNI sites, please visit the website: http://adni.loni.usc.edu/wp-content/themes/freshnews-dev-v2/documents/policy/ADNI_Acknowledgement_List%205-29-18.pdf. This study was also approved by the institutional review board of Taizhou First People’s Hospital. Authors cannot access to information that could identify individual participants during or after data collection.

### Measurement of CSF TNFα, TNFR1 and TNFR2 levels

The levels of CSF TNFα, TNFR1 and TNFR2 levels were measured at the Department of Neurology, Emory University. CSF TNFα, TNFR1 and TNFR2 levels were examined in duplicate. Commercially available multiplex immunoassays (Millipore Sigma, Burlington, MA) were utilized to examine the levels of CSF TNFα, TNFR1 and TNFR2. The inter-plate coefficients of variation (CV) of TNFα, TNFR1 and TNFR2 were 9.38%, 2.85% and 3.09%, respectively. Values were given in pg/ml.

### Statistical analysis

T test was performed to evaluate the differences in continuous variables (age, education, CDRSB, follow-up length, CSF TNFα, TNFR1 and TNFR2 levels), and x^2^ test was utilized to compare the distributions of categorical variables (APOE4 genotype and gender) between non-converters and converters. We categorized baseline CSF TNFα, TNFR1 and TNFR2 levels into tertiles. Associations of CSF TNFα, TNFR1 and TNFR2 levels with the incidence of dementia were evaluated using Cox proportional hazards regression analysis adjusted for age, gender, education, APOE4 genotype and CDRSB. CSF TNFα, TNFR1 and TNFR2 levels were treated as categorical variables in Cox proportional hazards regression models. All statistical work was performed using R (v. 4.0.2).

## Results

### Demographic and clinical data between non-converters and converters

The demographic and clinical characteristics of the study participants are demonstrated in Table 1. This study had a total of 129 MCI participants, including 54 non-converters and 75 converters. There were no differences in age, educational level or percentage of females between two groups. Compared to non-converters, converters had higher percentage of APOE4 carriers, higher CDRSB score, and longer follow-up time. However, there were no differences in levels of CSF TNFα, TNFR1 or TNFR2 between two groups (Table 1).

**Table 1:** Demographic and clinical information between non-converters and converters.

Variables	Non-converters (n = 54)	Converters (n = 75)	P value
Age, years	73.8 ± 7.98	74.8 ± 7.7	0.49
Education, years	15.6 ± 3.24	15.9 ± 2.8	0.59
Female gender, n (%)	22 (40.7)	26 (34.7)	0.48
APOE4 carriers, n (%)	22 (40.7)	48 (64)	0.009
CDRSB scores	1.25 ± 0.74	1.74 ± 0.94	0.001
Follow-up, years	3.07 ± 2.42	4.25 ± 2.63	0.01
CSF TNFα (pg/ml)	1.65 ± 0.61	1.77 ± 0.46	0.22
CSF TNFR1 (pg/ml)	894 ± 271	866 ± 221	0.12
CSF TNFR2 (pg/ml)	1058 ± 352	1053 ± 272	0.26

Abbreviations: CDRSB: Clinical Dementia Rating Sum of Boxes; TNFα: Tumor Necrosis Factor α; TNFR1: Tumor Necrosis Factor Receptor 1; TNFR2: Tumor Necrosis Factor Receptor 2.

### CSF TNFα levels predict conversion to dementia

We categorized CSF TNFα, TNFR1 and TNFR2 levels into tertiles respectively. For TNFα, this variable was categorized into three groups: low (0.21–1.53 pg/ml), intermediate (1.53–1.97 pg/ml) and high (1.97–3.32 pg/ml) groups. For TNFR1, this variable was categorized into three groups: low (382–744 pg/ml), intermediate (744–960 pg/ml) and high (960–1850 pg/ml) groups. For TNFR2, this variable was categorized into three groups: low (525–880 pg/ml), intermediate (880–1120 pg/ml) and high (1120–2310 pg/ml) groups.

**Fig 1:**
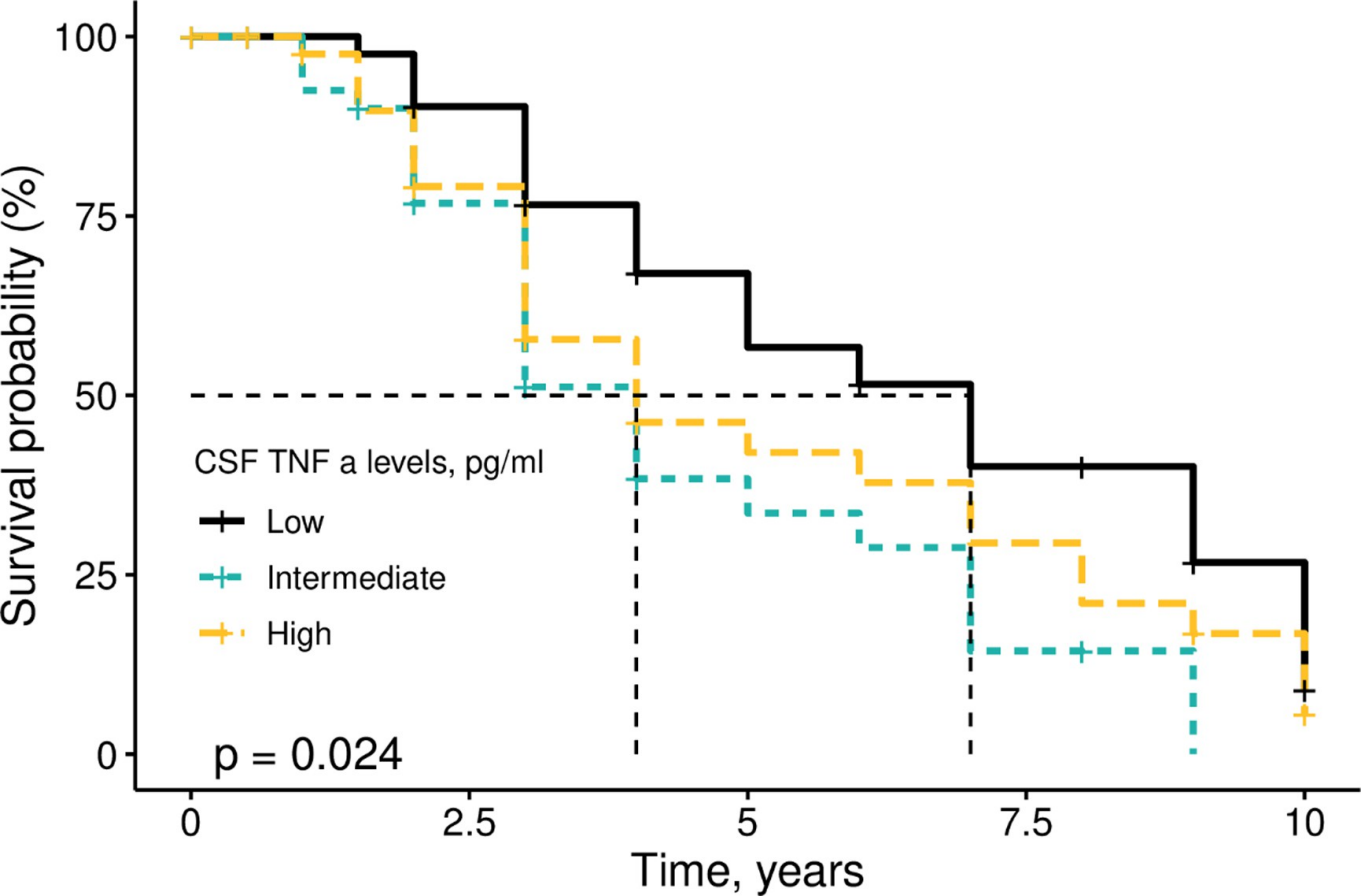
Survival curve for progression from MCI to dementia among participants with different CSF TNFα levels. CSF TNFα was categorized into three groups according to tertiles of its levels. There was a significant difference in rates of conversion to dementia between three groups (tertiles of TNFα levels) among MCI individuals (p = 0.024).

Kaplan-Meier analysis was used to display the associations of CSF TNFα, TNFR1 and TNFR2 levels with conversion to dementia among MCI individuals. As shown in survive curve (Fig 1), there was a significant difference in rates of conversion to dementia between three groups (tertiles of TNFα levels) among MCI individuals (p = 0.024). However, CSF TNFR1 and TNFR2 levels were not associated with conversion to dementia (S1 and S2 Figs; all p > 0.05).

**Fig 2:**
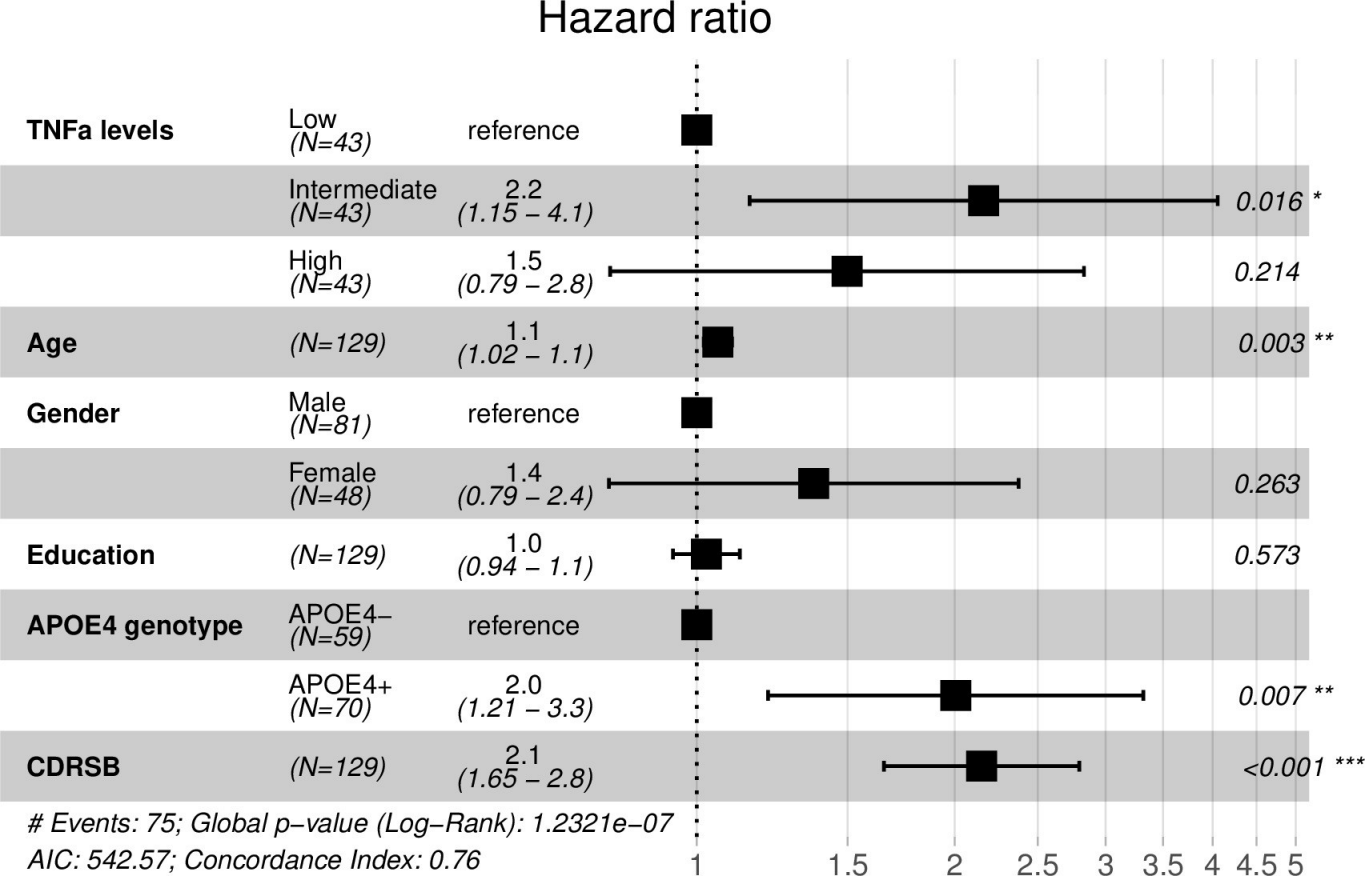
Summary of Cox proportional hazards regression model with CSF TNFα as the independent variable. Compared to the low group (the reference group), the intermediate group progressed more rapidly to dementia [HR (95% CI) = 2.2 (1.15–4.1); p = 0.016] after adjusting for other covariates. However, the high group did not progress faster than the low group [HR (95% CI) = 1.5 (0.79–2.8); p = 0.214].

To further examine whether CSF TNFα levels were associated with conversion to dementia among MCI older adults, Cox proportional hazards regression models were fitted after adjusting for age, gender, educational level, APOE4 genotype and CDRSB. As shown in Fig 2, we found that compared to the low group (the reference group), the intermediate group progressed more rapidly to dementia [HR (95% CI) = 2.2 (1.15–4.1); p = 0.016] after adjusting for other covariates. However, the high group did not progress faster than the low group [HR (95% CI) = 1.5 (0.79–2.8); p = 0.214].

## Discussion

To the best of our knowledge, this is the first study to investigate whether CSF TNFα levels are associated with the risk of conversion to dementia among MCI patients. Compared to the low group (the reference group), the intermediate group progressed more rapidly to dementia after adjusting for other covariates. However, the high group did not progress faster than the low group. The present study suggested a potential non-linear relationship between CSF TNFα levels and the risk of development of dementia.

Our finding that CSF TNFα was associated with the risk of incident dementia is in line with previous studies. For example, a cross-sectional study found increased levels of TNFα in affected brain regions of patients with AD dementia [12]. Levels of TNFα in blood and CSF were increased in patients with AD dementia or other forms of dementia [13–17]. Additionally, a previous study suggested that higher levels of TNFα in blood were associated with the risk of developing AD among cognitively normal community-dwelling older adults [7]. In contrast, Diniz and colleagues did not observe an association between serum TNFα levels and progression from MCI to AD during approximately 1.5 years of follow-up [8]. Consistent with this finding, Taipa and colleagues did not find an association of CSF TNFα levels with cognitive status at baseline and follow-up in AD patients [9]. These inconsistencies maybe due to the fact that the relationship between CSF TNFα levels and cognitive decline is actually non-linear. Our present study found that compared to the first tertile of CSF TNFα levels, the second tertile progressed faster to dementia while the third tertile did not, suggesting a potential non-linear relationship between CSF TNFα levels and the risk of development of dementia.

Our study has several limitations. First, the sample size of individuals whose CSF TNFα were < 1.3 pg/ml was relatively small. Further studies are needed to have sufficient sample size to increase the statistical power. Second, participants of the ANDI study were highly educated, which may limit our ability to generalize our findings to other population. Therefore, larger population-based cohorts are needed to replicate our results.

In conclusion, we found that high CSF TNFα levels were associated with the risk of conversion to dementia among MCI subjects. Our data highlight the importance of TNFα in the pathogenesis of AD and suggest that CSF TNFα could be used to predict subsequent conversion to dementia among MCI subjects.

## Supporting information

S1 Fig Survival curve for progression from MCI to dementia among participants with different CSF TNFR1 levels. CSF TNFR1 levels were categorized into three groups according to tertiles of its levels. CSF TNFR1 levels were not associated with conversion to dementia.
(TIF)

S2 Fig Survival curve for progression from MCI to dementia among participants with different CSF TNFR2 levels. CSF TNFR2 levels were categorized into three groups according to tertiles of its levels. CSF TNFR2 levels were not associated with conversion to dementia.
(TIF)
