# Supplementary figures and images for: CSF TNF α levels were associated with conversion from mild cognitive impairment to dementia
Source: PLoS One. 2022 Oct 26;17(10):e0274503. doi: 10.1371/journal.pone.0274503 (PMC9604923; doi:10.1371/journal.pone.0274503)

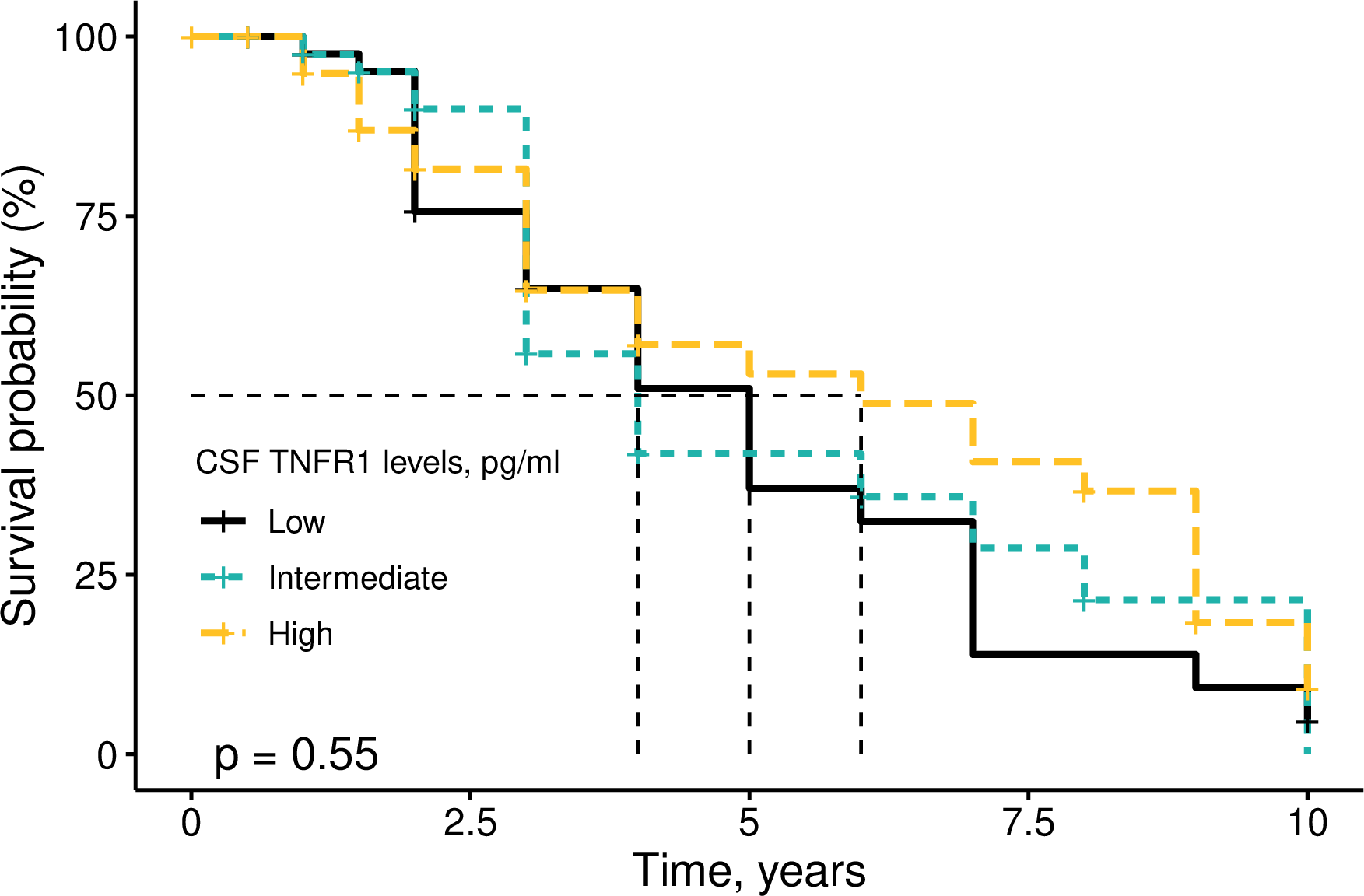

Supplement: S1 Fig — CSF TNFR1 levels were categorized into three groups according to tertiles of its levels. CSF TNFR1 levels were not associated with conversion to dementia. (TIF) [file pone.0274503.s001.tif]

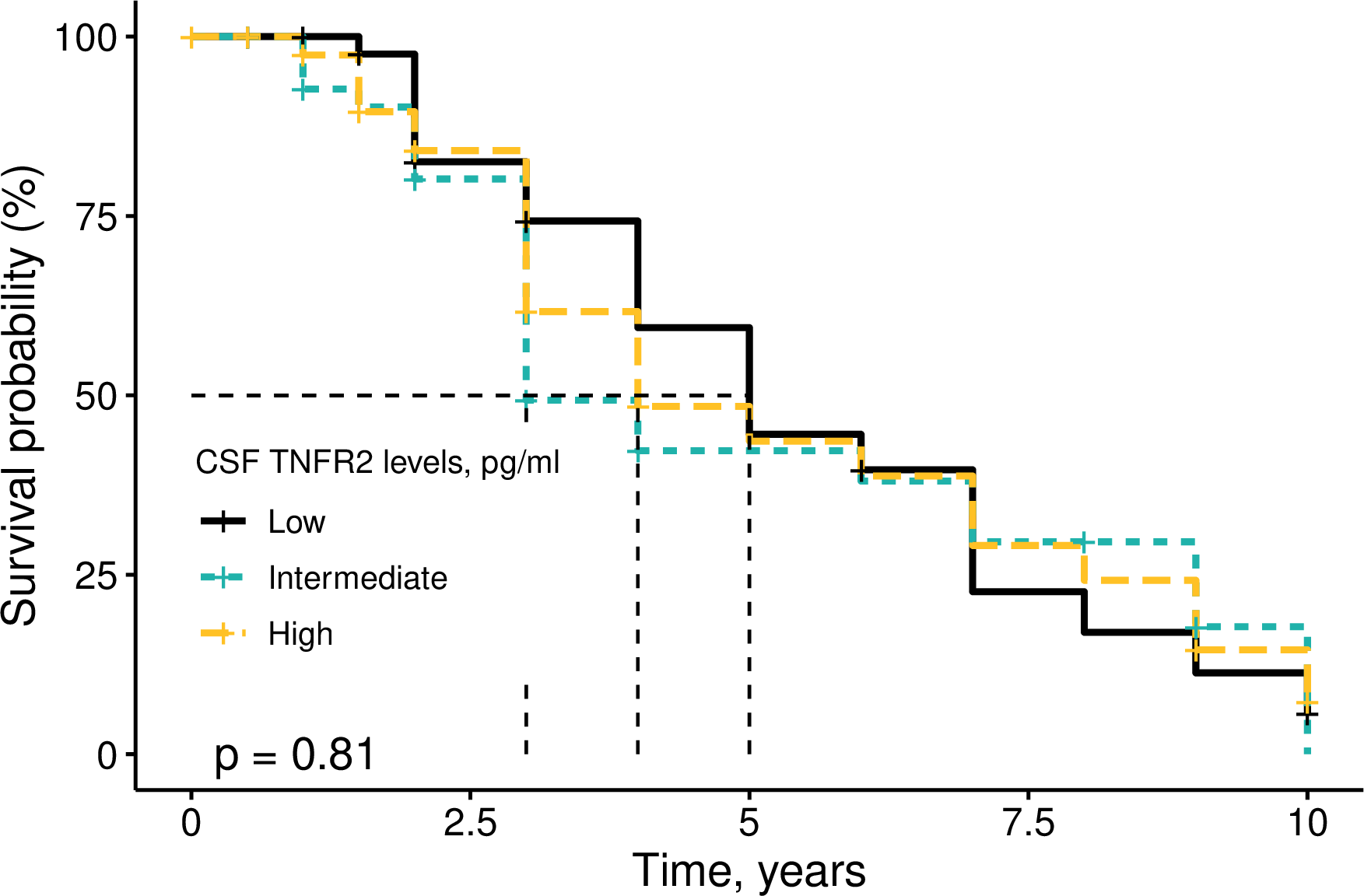

Supplement: S2 Fig — CSF TNFR2 levels were categorized into three groups according to tertiles of its levels. CSF TNFR2 levels were not associated with conversion to dementia. (TIF) [file pone.0274503.s002.tif]
